# Zinc deficiency predicts new-onset diabetic kidney disease in type 2 diabetes: a retrospective cohort study

**DOI:** 10.3389/fnut.2025.1653151

**Published:** 2025-09-24

**Authors:** Kuo-Chuan Hung, Ting-Sian Yu, Chih-Wei Hsu, Yi-Chen Lai, Ping-Heng Tan, Yao-Tsung Lin, Li-Kai Wang, Chien-Ming Lin, I-Wen Chen

**Affiliations:** ^1^Department of Anesthesiology, Chi Mei Medical Center, Tainan City, Taiwan; ^2^School of Medicine, College of Medicine, National Sun Yat-sen University, Kaohsiung City, Taiwan; ^3^Department of Anesthesiology, E-Da Hospital, I-Shou University, Kaohsiung City, Taiwan; ^4^Department of Psychiatry, Kaohsiung Chang Gung Memorial Hospital and Chang Gung University College of Medicine, Kaohsiung City, Taiwan; ^5^Department of Anesthesiology, Chi Mei Medical Center, Liouying, Tainan City, Taiwan

**Keywords:** micronutrient deficiency, trace elements, renal complications, mortality, chronic kidney disease, oxidative stress

## Abstract

**Background:**

Type 2 diabetes mellitus (T2DM) is a major global health challenge, with diabetic kidney disease (DKD) representing one of its most serious complications. Although zinc deficiency is common in diabetes, large-scale clinical evidence on its role as a predictor of new-onset DKD is limited.

**Methods:**

We conducted a retrospective cohort study using the TriNetX Analytics Network Platform to analyze patients aged ≥18 years who underwent zinc testing (2010–2023). Patients were classified into zinc deficiency (serum zinc <70 μg/dL) and control groups (70–120 μg/dL). After 1:1 propensity score matching, we analyzed the risk of new-onset DKD at two-year follow-up. The secondary outcomes included the risks of all-cause mortality, acute kidney injury (AKI), chronic kidney disease (CKD), poor glycemic control (HbA1c ≥ 7%), and ophthalmic complications.

**Results:**

The final matched cohort included 20,470 patients (10,235 per group) with a mean age of 54 years. Zinc deficiency was associated with a 42% increased risk of new-onset DKD (hazard ratio [HR] 1.42, 95% confidence interval [CI]: 1.20–1.68, *p* < 0.001). Additional significant associations included all-cause mortality (HR: 1.65, 95% CI: 1.40–1.95, *p* < 0.001), AKI (HR: 1.47, 95% CI: 1.27–1.69, *p* < 0.001), and CKD development (HR: 1.18, 95% CI: 1.02–1.37, *p* = 0.028). No significant associations were observed with poor glycemic control or ophthalmic complications. Subgroup analyses showed stronger associations in patients with diabetes duration <5 years (HR 1.65, 95% CI: 1.35–2.02, *p* < 0.001).

**Conclusion:**

Zinc deficiency is an independent predictor of new-onset DKD and adverse outcomes in T2DM, particularly in early disease. These findings support zinc deficiency as a potential biomarker for risk stratification and highlight the need for prospective studies to evaluate whether zinc supplementation can reduce risk.

## 1 Introduction

Diabetes mellitus is a chronic metabolic disease marked by hyperglycemia due to impaired insulin secretion or action. It is classified into type 1 (T1DM), an autoimmune form, and type 2 (T2DM), which accounts for over 90% of global cases (1). Strongly linked to obesity, sedentary lifestyle, and aging, T2DM continues to rise worldwide and represents the main driver of the public health and economic burden of diabetes (2–5). In 2017, an estimated 6.28% of the world’s population was affected, with projections indicating a continued increase through 2030 (6). Among these complications, diabetic kidney disease (DKD) has emerged as one of the most serious, developing in approximately 20–40% of patients with diabetes and serving as the leading cause of end-stage renal disease globally (7–9). The challenge for clinicians lies not only in managing established DKD, but also in identifying which patients among the large diabetic population will develop this complication (10–12). Early prediction of DKD onset would enable targeted monitoring and timely intervention and potentially improve outcomes through personalized care strategies. However, current clinical tools for predicting new-onset DKD remain limited, creating an urgent need for reliable biomarkers that can identify high-risk patients before clinical manifestations become apparent.

Zinc deficiency represents an underrecognized but potentially significant predictor of diabetic complications (13, 14), occurring with a markedly higher frequency in patients with diabetes than in the general population. Zinc is an essential trace element involved in numerous enzymatic reactions that maintain cellular integrity, immune function, and antioxidant defense (15). In diabetes, zinc deficiency may arise from increased urinary losses, impaired absorption, and altered tissue distribution (16, 17). These mechanisms are biologically relevant to kidney health, as zinc contributes to glomerular barrier stability, regulation of inflammation, and protection against oxidative stress (18–21). Despite the biological plausibility of zinc deficiency as a predictive biomarker for DKD, prior studies have primarily employed cross-sectional (22–26) or interventional designs (27, 28), but none have addressed zinc deficiency as a predictor of DKD using longitudinal, real-world data. We therefore conducted a retrospective cohort study using the TriNetX Analytics Network to test the hypothesis that zinc deficiency predicts new-onset DKD in patients with T2DM. We further examined secondary renal and mortality outcomes, as well as potential effect modification across clinically relevant subgroups.

## 2 Methods

### 2.1 Data sources

This retrospective study utilized data from the TriNetX Analytics Network Platform, a federated health research network that aggregates electronic health records from healthcare organizations worldwide. The platform provides access to real-world clinical data while maintaining patient privacy through a secure Health Insurance Portability and Accountability Act-compliant infrastructure. The dataset incorporates diverse clinical variables, including demographic information, laboratory values, diagnoses coded using the International Classification of Diseases, Tenth Revision, Clinical Modification (ICD-10-CM), procedures, and prescribed medications with standardized drug coding systems. The TriNetX database has been widely adopted in clinical research and its reliability is well supported by numerous peer-reviewed studies (29–31). The study was approved by the Institutional Review Board of Chi Mei Medical Center (IRB number: 11310-E04). Informed consent was not required for this retrospective study, as it involved only secondary analysis of de-identified data from the TriNetX platform, with no access to personal or identifiable patient information.

### 2.2 Study population and eligibility criteria

We identified patients aged 18 years and older who underwent zinc testing between January 1, 2010, and January 31, 2023. Based on serum zinc levels, patients were classified into two groups using clinically established thresholds: the zinc deficiency group (ZD group) with serum zinc levels below 70 μg/dL and the control group with serum zinc levels between 70 and 120 μg/dL. Patients with zinc levels > 120 μg/dL were excluded to avoid potential confounding from zinc supplementation or toxicity. Zinc deficiency was defined as serum zinc <70 μg/dL, in line with cut-offs used in prior study (32). The date of zinc testing served as the index date for each patient, establishing a clear temporal reference point for outcome assessment. All eligible patients were required to have an established diagnosis of T2DM prior to the index date, ensuring that our study population consisted of patients with pre-existing diabetes who subsequently developed or did not develop renal complications.

### 2.3 Exclusion criteria

Exclusion criteria were applied to ensure a homogeneous study population and minimize confounding factors. We excluded patients with a history of chronic kidney disease or acute kidney injury (AKI) prior to the index date. Additionally, patients with pre-existing DKD before the index date were excluded. We also excluded patients with a history of hemodialysis based on procedure codes, nephritic syndrome, hypertensive chronic kidney disease, contrast-induced nephropathy, HIV infection, kidney transplant status, malignant neoplasms of the urinary tract, and severe anemia (i.e., hemoglobin level ≤8 mg/dL) documented before the index date.

### 2.4 Data collection and matching strategy

In the current study, we employed a 1:1 propensity score-matching approach to minimize confounding variables and ensure comparability between groups. Matching was extended beyond basic demographic and clinical variables to incorporate detailed laboratory and therapeutic data. Baseline characteristics were extracted from the three-year period preceding the index date and included age, sex, race, body mass index (BMI), estimated glomerular filtration rate (eGFR), serum albumin, hemoglobin A1c (HbA1c), and hemoglobin levels. Patients with incomplete baseline information required for propensity score matching were excluded; no imputation was performed. To minimize confounding by diabetes severity, we matched patients for the presence of diabetes-related complications such as diabetic ketoacidosis and ophthalmic, neurological, and circulatory complications. Furthermore, we controlled for the use of second-line antidiabetic agents, specifically glucagon-like peptide-1 receptor agonists (GLP-1 RAs) and sodium-glucose cotransporter-2 inhibitors (SGLT2is), owing to their recognized renoprotective effects independent of glycemic control. To further address potential treatment bias, we matched for zinc supplementation use, as well as the use of angiotensin-converting enzyme (ACE) inhibitors and angiotensin II receptor blockers (ARBs).

### 2.5 Study outcomes

The primary outcome was the development of new-onset DKD at two-year follow-up, defined using ICD-10-CM code E11.2. Secondary outcomes included two-year all-cause mortality, incident AKI (defined by ICD-10-CM codes N17), chronic kidney disease (defined by ICD-10-CM codes N18) development, high HbA1c levels (i.e., ≥7%), and T2DM-related ophthalmic complications.

HbA1c level was included as a secondary outcome to determine whether observed renal complications are mediated through glycemic control or represent a direct effect of zinc deficiency. T2DM-related ophthalmic complications were used as negative control outcomes to test the specificity of the hypothesis. Since both nephropathy and retinopathy are microvascular complications of diabetes, using ophthalmic complications as a negative control helps determine whether zinc deficiency specifically affects renal microvascular function or broadly affects diabetic microvascular complications. To examine the early effects of zinc deficiency and capture the temporal relationship between exposure and outcome, we also analyzed outcomes at one-year follow-up. To reduce outcome misclassification and avoid including prevalent cases misidentified as incident cases, we implemented a one-month washout period, excluding any outcomes occurring within the first month after the index date.

### 2.6 Subgroup analyses

To explore potential effect modifications, we performed prespecified subgroup analyses stratified by clinically relevant characteristics. These included age groups (18–50 years versus >50 years), sex, presence of lipid disorders, hypertension status, HbA1c levels (<7% versus ≥7%), anemia status, duration of T2DM, and use of GLP-1 RAs/SGLT2is. These subgroup analyses were designed to identify patient populations who might be at a particularly high risk for zinc deficiency-related renal complications.

### 2.7 Statistical analysis

Baseline characteristics were summarized using appropriate descriptive statistics, with continuous variables presented as means with standard deviations (SD) and categorical variables as frequencies and percentages. To balance the baseline characteristics between the zinc deficiency and control groups, we implemented propensity score matching using a greedy nearest-neighbor algorithm. The quality of matching was assessed using standardized mean differences (SMD) and visual inspection of propensity score distributions. Time-to-event outcomes were analyzed using the Kaplan–Meier method, with between-group differences assessed via the log-rank test. The association between zinc status and clinical outcomes was quantified using Cox proportional hazards regression models to calculate hazard ratios (HRs) with 95% confidence intervals (CIs). The proportional hazard assumption was tested using Schoenfeld residuals.

For subgroup analyses, the statistical significance of differences between subgroups was evaluated by examining confidence interval overlap, a conservative approach that reduces the likelihood of false-positive findings. The age subgroups (18–50 years versus >50 years) were selected for exploratory analysis to assess potential effect modification across a broad age range; this cut-off was not based on a known biological threshold for zinc metabolism or DKD risk but reflects commonly used categories in epidemiologic research. All statistical analyses were conducted using the TriNetX Analytics Platform (TriNetX, Cambridge, MA, United States; https://trinetx.com), which provides built-in statistical tools for propensity score matching, Kaplan–Meier estimation, Cox proportional hazards modeling, and calculation of HRs with 95% CIs. A two-sided *p*-value of <0.05 was considered statistically significant.

## 3 Result

### 3.1 Patient selection and baseline characteristics

**Figure 1:**
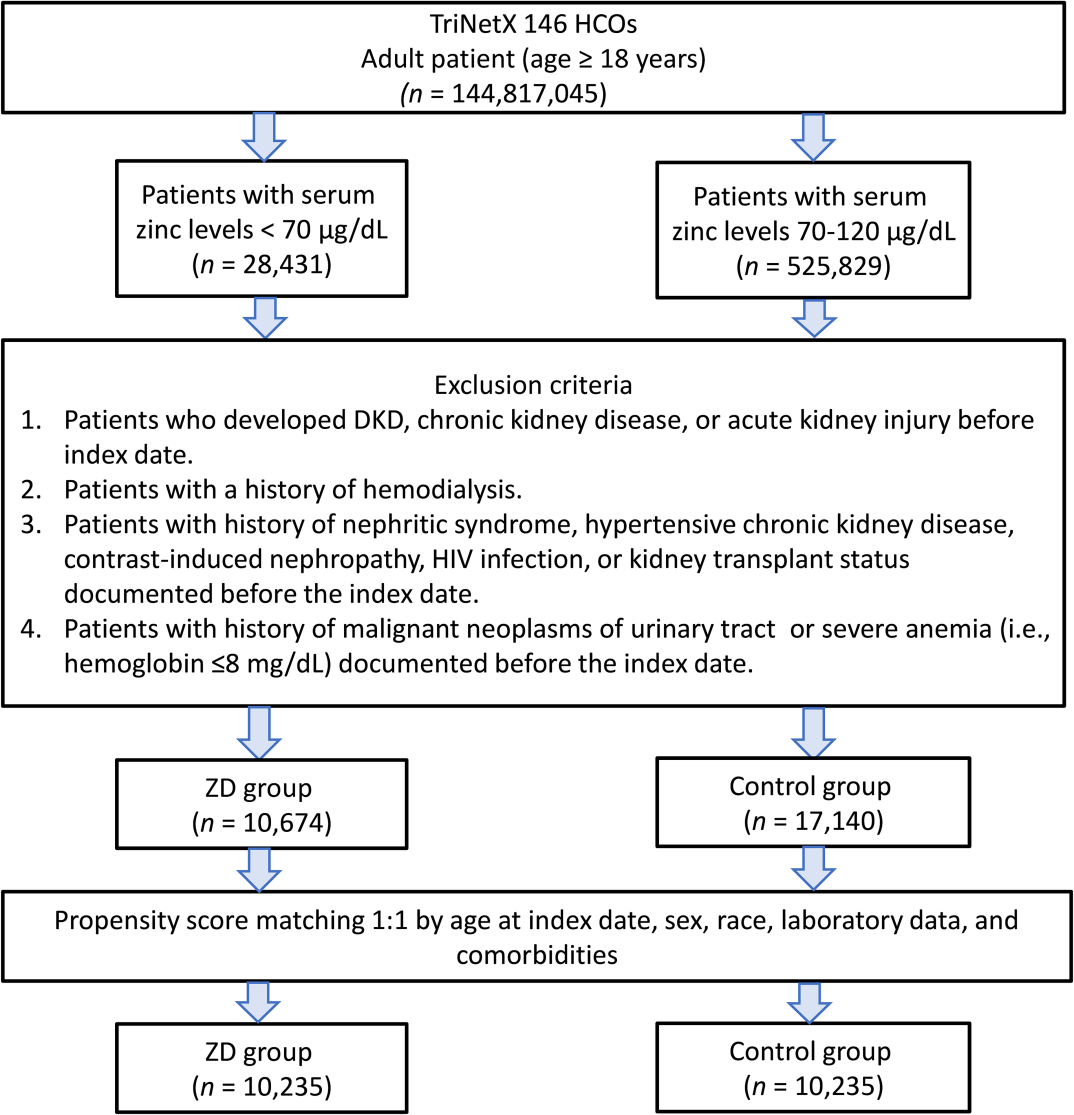
Patient selection flowchart from the TriNetX database. The flowchart illustrates the systematic exclusion process applied to identify eligible patients with zinc deficiency (ZD) and zinc sufficiency (control group). HCOs, Healthcare Organizations; DKD, diabetic kidney disease.

**Figure 2:**
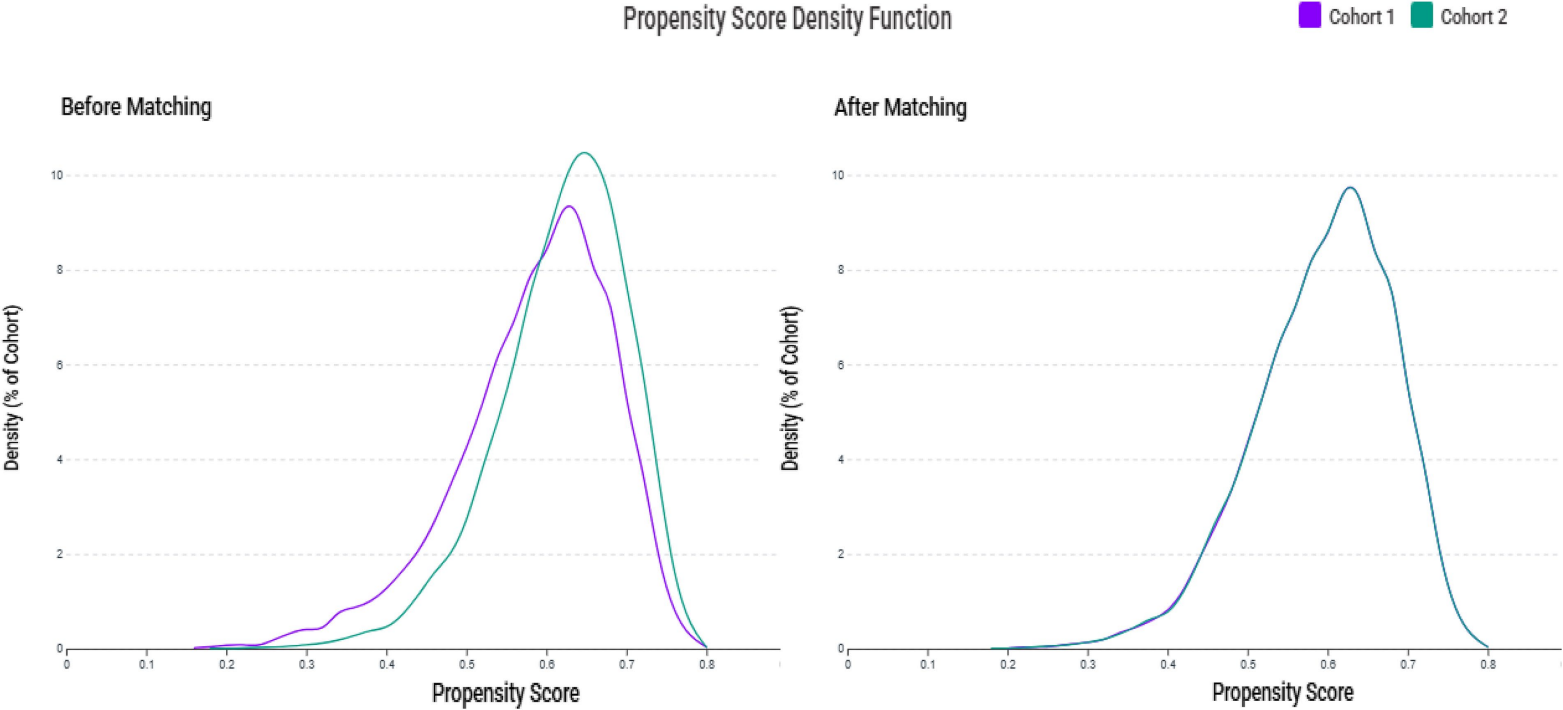
Propensity score density distributions before and after matching. The left panel illustrates the differing distribution patterns between the zinc deficiency group (Cohort 1) and the control group (Cohort 2) prior to matching. The right panel displays the improved overlap and balance achieved through 1:1 matching with a caliper of 0.1 standard deviations.

We initially identified 554,260 patients who underwent zinc testing (Figure 1). Following the application of the inclusion and exclusion criteria, we successfully included 10,674 patients with zinc deficiency and 17,140 patients with normal zinc levels in the pre-matching cohort. After implementing 1:1 matching, we obtained a final cohort of 20,470 patients (10,235 in each group). The propensity score distributions demonstrated excellent overlap after matching (Figure 2).

The matched cohort had several important demographic characteristics (Table 1). The patients averaged 54 years of age, with approximately two-thirds being female. The high prevalence of comorbidities was striking, with hypertension affecting nearly 65% of the patients and obesity affecting over 60%. The prevalence of existing diabetic complications was relatively low, with neurological complications in approximately 14% and ophthalmic complications in only around 4% of patients, suggesting that our cohort captured patients in earlier stages of diabetic disease progression. Laboratory parameters after matching revealed that both groups had preserved kidney function, with mean estimated glomerular filtration rates exceeding 90 mL/min/1.73 m^2^. The similar distribution of renoprotective medications, including ACE inhibitors, ARBs, GLP-1 receptor agonists, and SGLT-2 inhibitors, between groups strengthens our ability to attribute observed differences to zinc status rather than differential medical management.

**Table 1:** Baseline characteristics of patients before and after propensity score matching.

Variables	Before matching			After matching		
	ZD group (*n* = 10,674)	Control group (*n* = 17,140)	SMD^†^	ZD group (*n* = 10,235)	Control group (*n* = 10,235)	SMD^†^
Patient characteristics						
Age at index (years)	54.2 ± 15.0	53.6 ± 14.4	0.043	54.0 ± 14.9	54.0 ± 14.5	0.001
Female	7,062 (66.2%)	11,325 (66.1%)	0.002	6,837 (66.8%)	6,851 (66.9%)	0.003
BMI kg/m^2^	34.7 ± 10.1	35.4 ± 9.4	0.080	35.0 ± 10.0	35.0 ± 9.6	0.005
White	6,081 (57.0%)	10,012 (58.4%)	0.029	5,835 (57.0%)	5,834 (57.0%)	<0.001
Black or African American	2,105 (19.7%)	3,008 (17.6%)	0.056	2008 (19.6%)	1987 (19.4%)	0.005
Unknown Race	1,684 (15.8%)	2,837 (16.6%)	0.021	1,617 (15.8%)	1,654 (16.2%)	0.010
Other Race	545 (5.1%)	879 (5.1%)	0.001	528 (5.2%)	527 (5.1%)	<0.001
Asian	167 (1.6%)	286 (1.7%)	0.008	161 (1.6%)	161 (1.6%)	<0.001
Comorbidities						
Essential (primary) hypertension	6,893 (64.6%)	11,176 (65.2%)	0.013	6,609 (64.6%)	6,572 (64.2%)	0.008
Overweight and obesity	6,535 (61.2%)	11,014 (64.3%)	0.063	6,409 (62.6%)	6,409 (62.6%)	<0.001
Dyslipidemia	5,650 (52.9%)	10,108 (59.0%)	0.122	5,506 (53.8%)	5,490 (53.6%)	0.003
Vitamin D deficiency	3,726 (34.9%)	6,152 (35.9%)	0.021	3,609 (35.3%)	3,615 (35.3%)	0.001
Neoplasms	2,937 (27.5%)	4,814 (28.1%)	0.013	2,794 (27.3%)	2,808 (27.4%)	0.003
Diseases of liver	2,253 (21.1%)	3,180 (18.6%)	0.064	2078 (20.3%)	2065 (20.2%)	0.003
Anemias	1952 (18.3%)	2,536 (14.8%)	0.094	1777 (17.4%)	1759 (17.2%)	0.005
Ischemic heart diseases	1,614 (15.1%)	2,223 (13.0%)	0.062	1,474 (14.4%)	1,470 (14.4%)	0.001
Nicotine dependence	1,229 (11.5%)	1,647 (9.6%)	0.062	1,094 (10.7%)	1,097 (10.7%)	0.001
Cerebrovascular diseases	899 (8.4%)	1,112 (6.5%)	0.074	792 (7.7%)	790 (7.7%)	0.001
COVID-19	799 (7.5%)	1,099 (6.4%)	0.042	746 (7.3%)	731 (7.1%)	0.006
Malnutrition	992 (9.3%)	903 (5.3%)	0.155	740 (7.2%)	783 (7.7%)	0.016
Heart failure	818 (7.7%)	947 (5.5%)	0.086	708 (6.9%)	714 (7.0%)	0.002
Alcohol related disorders	551 (5.2%)	488 (2.8%)	0.118	402 (3.9%)	433 (4.2%)	0.015
Other rheumatoid arthritis	385 (3.6%)	552 (3.2%)	0.021	365 (3.6%)	381 (3.7%)	0.008
Gout	296 (2.8%)	483 (2.8%)	0.003	284 (2.8%)	276 (2.7%)	0.005
Systemic lupus erythematosus (SLE)	134 (1.3%)	178 (1.0%)	0.020	124 (1.2%)	132 (1.3%)	0.007
T2DM with neurological complications	1,502 (14.1%)	2091 (12.2%)	0.055	1,392 (13.6%)	1,379 (13.5%)	0.004
T2DM with ophthalmic complications	414 (3.9%)	632 (3.7%)	0.010	388 (3.8%)	404 (3.9%)	0.008
T2DM with circulatory complications	400 (3.7%)	546 (3.2%)	0.031	366 (3.6%)	349 (3.4%)	0.009
T2DM with ketoacidosis	105 (1.0%)	106 (0.6%)	0.041	83 (0.8%)	92 (0.9%)	0.010
Laboratory data						
Hemoglobin≥12 mg/dL	8,491 (79.5%)	13,837 (80.7%)	0.030	8,167 (79.8%)	8,207 (80.2%)	0.010
Hemoglobin A1c ≥ 7%	3,523 (33.0%)	6,035 (35.2%)	0.047	3,381 (33.0%)	3,444 (33.6%)	0.013
Albumin g/dL (≥3.5 g/dL)	8,485 (79.5%)	14,044 (81.9%)	0.062	8,209 (80.2%)	8,240 (80.5%)	0.008
eGFR>60 mL/min/1.73 m^2^	91.8 ± 29.9	90.2 ± 26.6	0.056	91.1 ± 29.0	90.9 ± 27.4	0.009
Medications						
Antilipemic agents	4,191 (39.3%)	7,204 (42.0%)	0.056	4,032 (39.4%)	4,032 (39.4%)	<0.001
Insulins and analogues	4,326 (40.5%)	6,079 (35.5%)	0.104	4,014 (39.2%)	4,029 (39.4%)	0.003
ACE inhibitors	2,477 (23.2%)	4,080 (23.8%)	0.014	2,373 (23.2%)	2,368 (23.1%)	0.001
Angiotensin II inhibitor	1776 (16.6%)	3,188 (18.6%)	0.051	1730 (16.9%)	1760 (17.2%)	0.008
GLP-1analogues	1,603 (15.0%)	2,818 (16.4%)	0.039	1,586 (15.5%)	1,548 (15.1%)	0.010
SGLT2 inhibitors	628 (5.9%)	1,243 (7.3%)	0.055	616 (6.0%)	613 (6.0%)	0.001
Zinc supplementation	405 (3.8%)	597 (3.5%)	0.017	378 (3.7%)	390 (3.8%)	0.006

VDD, vitamin D deficiency; BMI, body mass index; SMD, standardized mean differences; † SMD values <0.1 indicate adequate balance between groups; eGFR, estimated Glomerular Filtration Rate; COPD, chronic obstructive pulmonary disease; GLP-1, Glucagon-like peptide-1; SGLT2, Sodium-glucose co-transporter 2; T2DM, Type 2 diabetes mellitus.

### 3.2 Primary outcome: two-year clinical outcomes

**Figure 3:**
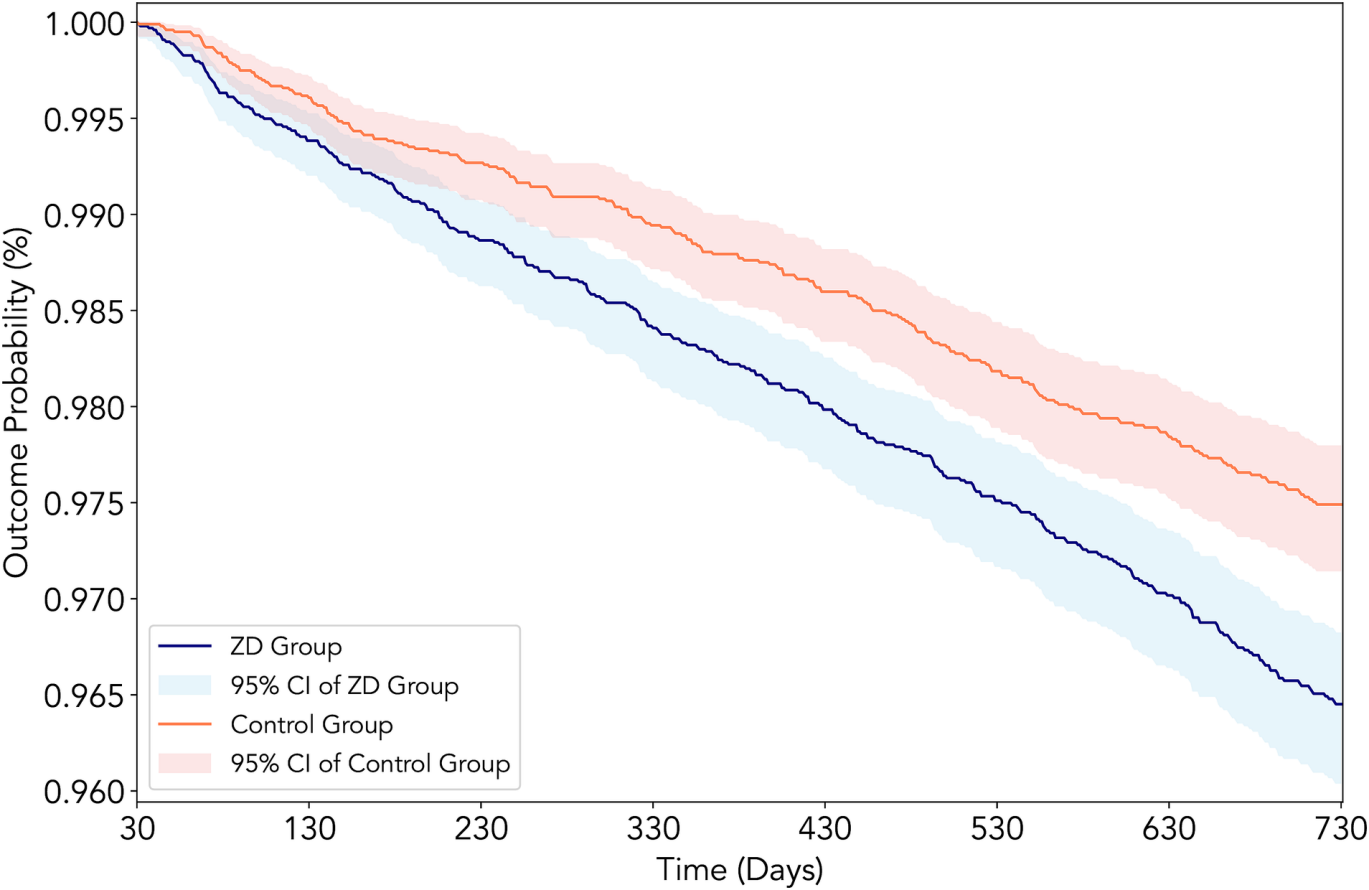
Kaplan–Meier survival curves for new-onset diabetic kidney disease (DKD) in patients with type 2 diabetes mellitus according to zinc status. The curves depict the cumulative incidence of DKD over 2 years of follow-up in the propensity score-matched cohort, comparing patients with zinc deficiency (serum zinc <70 μg/dL, red line) versus those with normal zinc levels (70–120 μg/dL, blue line). The log-rank test indicated a significantly higher incidence of DKD in the zinc deficiency group (hazard ratio: 1.42, 95% CI: 1.20–1.68, *p* < 0.001).

The primary analysis revealed evidence for zinc deficiency as a significant risk factor for DKD development over 2 years of follow-up (Table 2). New-onset DKD was observed in 313 patients in the zinc deficiency group versus 229 in the control group, reflecting a 42% higher risk associated with zinc deficiency (HR 1.42, 95% CI: 1.20–1.68, *p* < 0.001, risk difference: 0.82%) (Figure 3). Beyond the primary endpoint, zinc deficiency showed broad association with adverse clinical outcomes. All-cause mortality showed the most pronounced effect, with zinc-deficient patients experiencing a 65% increased risk of death (HR 1.65, 95% CI: 1.40–1.95, *p* < 0.001). AKI events were similarly higher, affecting 4.6% of zinc-deficient patients versus 3.2% of controls (HR 1.47, 95% CI: 1.27–1.69, *p* < 0.001).

**Table 2:** Association between zinc deficiency and 2-year outcomes.

Outcomes	ZD group (*n* = 10,235)	Control group (*n* = 10,235)	HR (95% CI)	*p*-value
	Events (%)	Events (%)		
DKD	313 (3.1%)	229 (2.2%)	1.42 (1.20–1.68)	<0.001
Mortality	367 (3.6%)	230 (2.2%)	1.65 (1.40–1.95)	<0.001
AKI	468 (4.6%)	332 (3.2%)	1.47 (1.27–1.69)	<0.001
CKD	379 (3.7%)	333 (3.3%)	1.18 (1.02–1.37)	0.028
Hemoglobin A1c ≥ 7%	2,133 (20.8%)	2,266 (22.1%)	0.96 (0.91–1.02)	0.223
T2DM with ophthalmic complications	390 (3.8%)	363 (3.5%)	1.11 (0.96–1.28)	0.157

DKD, diabetic kidney disease; T2DM, Type 2 diabetes mellitus; CKD, chronic kidney disease; AKI, acute kidney injury; ZD, zinc deficiency; HR, hazard ratio; CI, confidence interval.

Chronic kidney disease development showed a small but statistically significant association (HR 1.18, 95% CI: 1.02–1.37, *p* = 0.028). The modest association observed between zinc deficiency and chronic kidney disease should be interpreted cautiously, as it may partly reflect coding overlap with DKD rather than an independent effect. Regarding glycemic control, there was no significant difference in the incidence of poor glycemic control (i.e., HbA1c levels ≥7%) between groups (HR 0.96, 95% CI: 0.91–1.02, *p* = 0.223), suggesting a direct mechanistic relationship between zinc status and renal function rather than an indirect effect mediated through worsened diabetes control. Similarly, diabetic ophthalmic complications showed no significant association (HR 1.11, 95% CI: 0.96–1.28, *p* = 0.157), supporting the specificity of the impact of zinc deficiency on renal rather than other microvascular complications.

### 3.3 Short-term outcomes at one year

The one-year analysis revealed that most zinc deficiency-related complications emerged early (Table 3). Zinc deficiency was associated with a significantly higher risk of DKD at 1 year (HR 1.44, 95% CI: 1.13–1.82, *p* = 0.003), a finding that remained consistent at the two-year mark (HR 1.42). Short-term mortality risk was markedly higher, with a 76% increase (HR 1.76, 95% CI: 1.42–2.18, *p* < 0.001). During the one-year follow-up, the proportional hazards assumption was satisfied (*p* = 0.466), indicating that the increased mortality risk associated with zinc deficiency remained consistent over time rather than being confined to the early period. AKI maintained its strong association at 1 year (HR 1.53, 95% CI: 1.28–1.84, *p* < 0.001), while chronic kidney disease showed no significant association (HR 1.11, 95% CI: 0.91–1.36, *p* = 0.295). This pattern may reinforce the concept that zinc deficiency primarily affects acute renal complications and diabetes-specific nephropathy rather than the gradual development of chronic kidney disease from other causes. In addition, the delayed significance in chronic kidney disease may also reflect either cumulative exposure effects or delayed coding capture in real-world databases.

**Table 3:** Association between zinc deficiency and 1-year outcomes.

Outcomes	ZD group (*n* = 10,235)	Control group (*n* = 10,235)	HR (95% CI)	*p*-value
	Events (%)	Events (%)		
DKD	163 (1.6%)	117 (1.1%)	1.44 (1.13–1.82)	0.003
Mortality	226 (2.2%)	132 (1.3%)	1.76 (1.42–2.18)	<0.001
AKI	291 (2.8%)	196 (1.9%)	1.53 (1.28–1.84)	<0.001
CKD	204 (2.0%)	189 (1.8%)	1.11 (0.91–1.36)	0.295
Hemoglobin A1c > 7%	1,624 (15.9%)	1708 (16.7%)	0.97 (0.91–1.04)	0.433
T2DM with ophthalmic complications	274 (2.7%)	239 (2.3%)	1.18 (0.99–1.40)	0.064

DKD, diabetic kidney disease; T2DM, Type 2 diabetes mellitus; CKD, chronic kidney disease; AKI, acute kidney injury; ZD, zinc deficiency; HR, hazard ratio; CI, confidence interval.

### 3.4 Subgroup analyses

**Figure 4:**
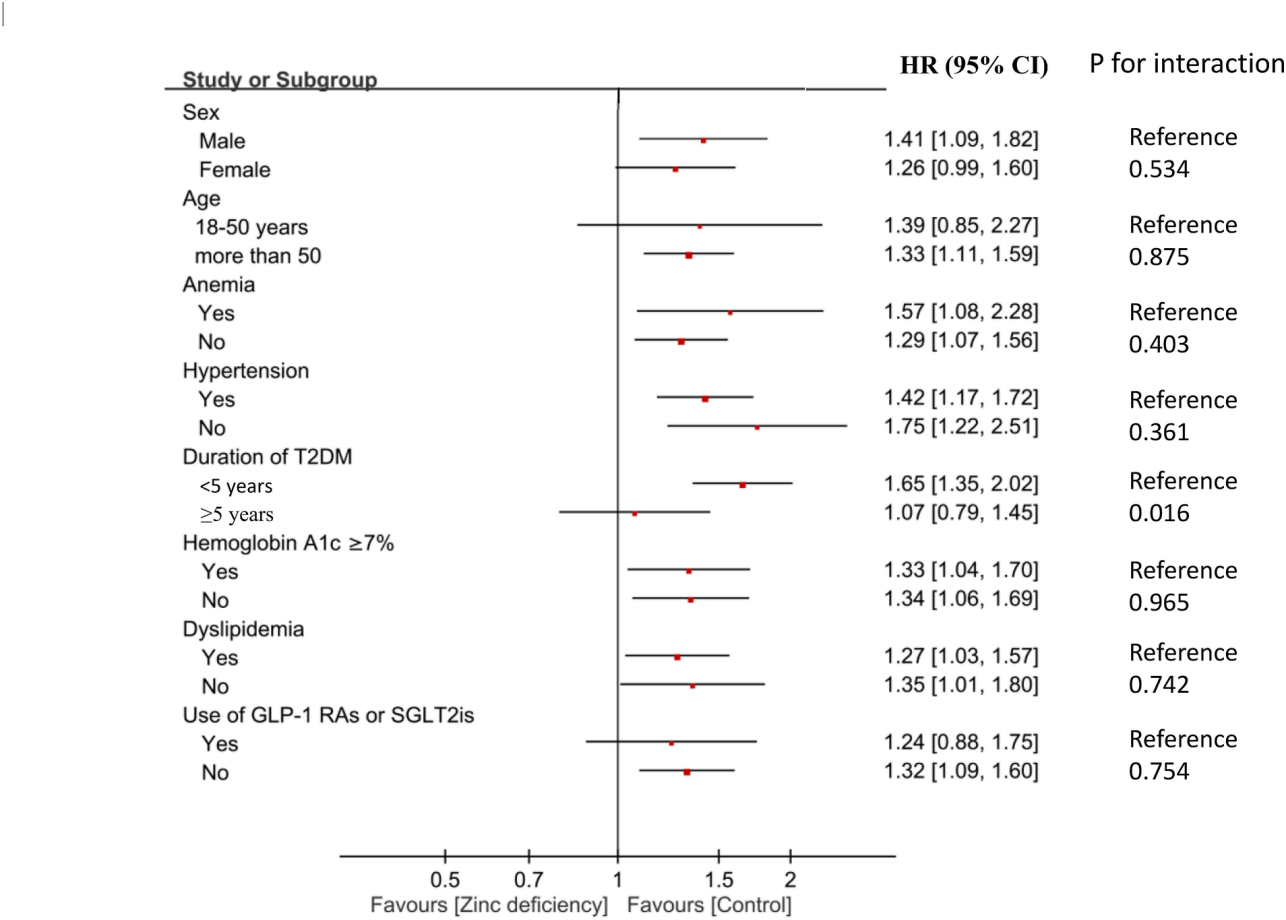
Forest plot showing subgroup analyses of association between zinc deficiency and risk of diabetic kidney disease (DKD) at 2-Year Follow-Up. HR, hazard ratio; CI, confidence interval; T2DM, Type 2 diabetes mellitus; GLP-1 Ras, glucagon-like peptide-1 receptor agonists; SGLT2is, sodium-glucose cotransporter-2 inhibitors.

In subgroup analyses, diabetes duration emerged as a significant effect modifier (*p* for interaction = 0.016) (Figure 4; Supplementary Table 1). Patients with a diabetes duration of less than 5 years showed a pronounced association between zinc deficiency and DKD (HR 1.65, 95% CI: 1.35–2.02, *p* < 0.001), while those with a longer diabetes duration showed no significant association (HR 1.07, 95% CI: 0.79–1.45, *p* = 0.667). This finding suggests that zinc deficiency may be particularly harmful during the early phases of diabetic disease progression. Patients with or without anemia, hypertension, dyslipidemia, GLP-1 receptor agonist/SGLT2 inhibitor use, or poor glycemic control showed comparable associations between zinc deficiency and the risk of developing DKD (all *p* for interaction>0.05). For non-significant subgroups, wide confidence intervals suggest limited statistical power, and these findings should be interpreted cautiously given the potential for type II error. This consistency suggests that zinc deficiency is an independent risk factor that operates across diverse clinical presentations, rather than being confined to specific patient subtypes.

### 3.5 Multivariable analysis of risk factor for diabetic kidney disease

Multivariable analysis confirmed zinc deficiency as an independent predictor of DKD development after controlling for all measured confounding factors (Table 4) (HR,1.45;95% CI: 1.26–1.67, *p* < 0.001). In addition, heart failure emerged as the strongest predictor (HR: 1.90, 95% CI: 1.54–2.35, *p* < 0.001), underscoring the relative magnitude of zinc deficiency in comparison with established risk factors. Interestingly, liver disease also emerged as a significant predictor (HR 1.59, 95% CI: 1.35–1.87, *p* < 0.001), possibly reflecting shared pathophysiological pathways or the liver’s role in zinc metabolism and storage. In contrast, overweight and obesity showed a protective association (HR 0.76, 95% CI: 0.64–0.89, *p* < 0.001). This paradoxical finding might reflect the “obesity paradox” observed in chronic diseases (33), where moderate obesity may provide metabolic reserves during illness.

**Table 4:** Risk factors for new-onset diabetic kidney disease (DKD).

Variable	HR (95% CI)	*p* value^†^
Zinc deficiency vs. control group	1.45 (1.26, 1.67)	<0.001
Male	1.66 (1.43, 1.93)	<0.001
Age at Index	1.03 (1.03, 1.04)	<0.001
Anemias	1.01 (0.84, 1.22)	0.893
Heart failure	1.90 (1.54, 2.35)	<0.001
Dyslipidemia	1.06 (0.90, 1.25)	0.495
Essential (primary) hypertension	1.45 (1.20, 1.74)	<0.001
Overweight and obesity	0.76 (0.64, 0.89)	<0.001
Nicotine dependence	1.12 (0.89, 1.40)	0.333
Ischemic heart diseases	1.18 (0.99, 1.42)	0.072
Neoplasms	0.99 (0.85, 1.16)	0.889
Diseases of liver	1.59 (1.35, 1.87)	<0.001
COVID-19	0.96 (0.72, 1.29)	0.800

HR, hazard ratio; CI, confidence interval; † multivariate logistic regression analysis.

## 4 Discussion

This study demonstrated that zinc deficiency serves as an independent predictor of new-onset DKD in patients with T2DM. In addition, zinc-deficient individuals have a significantly higher risk of multiple adverse outcomes, with the most pronounced effects observed for all-cause mortality and AKI. The association between zinc deficiency and DKD remained consistent across both the short- and long-term follow-up periods, suggesting a persistent rather than progressive risk pattern. Importantly, the observed effects appeared to be mediated through direct renal mechanisms rather than through poor glycemic control, as evidenced by the lack of association with worsened HbA1c levels and the specificity for renal rather than other microvascular complications. These findings establish zinc deficiency as a potentially valuable biomarker for risk stratification in diabetic populations, particularly among patients in earlier stages of disease progression.

In the current study, the consistency of risk elevation across both one-year and two-year follow-up periods suggests that zinc deficiency confers immediate and sustained vulnerability to renal complications rather than requiring prolonged exposure for effect manifestation. Our findings provide several novel insights. First, it represents the largest examination of zinc status as a predictor of incident DKD, moving beyond cross-sectional studies that cannot establish temporal relationships. Second, our use of real-world electronic health record data spanning over a decade provides external validity that smaller controlled studies cannot achieve. Third, the observation that zinc deficiency exerts early and sustained effects suggests that micronutrient deficiencies may act as immediate rather than progressively accumulating risk factors. Current guidelines for DKD prevention focus primarily on glycemic control, blood pressure management, and the use of renoprotective medications (34). Our results suggest that zinc status assessment could enhance risk stratification algorithms, potentially identifying high-risk patients who would benefit from intensified monitoring and early intervention before clinical manifestations become apparent.

Mechanistic insights from our analysis support a relationship between zinc deficiency and DKD. The absence of significant associations with diabetic ophthalmic complications suggests that zinc deficiency affects renal function through direct mechanisms rather than through broad microvascular dysfunction. Our observation that glycemic control outcomes were similar between zinc-deficient and zinc-sufficient patients provides crucial evidence that the observed renal effects are not mediated by worsened diabetes management. This distinction is clinically important because it suggests that zinc deficiency represents an independent therapeutic target rather than simply a marker of overall diabetes severity. This specificity is biologically plausible, given the critical role of zinc in maintaining glomerular barrier function and supporting antioxidant defense systems that protect against hyperglycemia-induced renal damage (18–21).

The demonstration of higher AKI risk associated with zinc deficiency represents a novel finding with important clinical implications. The robust association observed at both one-year and two-year follow-up suggests that zinc deficiency creates sustained vulnerability to acute renal insults. This finding extends beyond DKD and suggests that zinc deficiency may impair the ability of the kidney to respond to various stressors, including medications, procedures, and intercurrent illnesses commonly encountered in patients with diabetes. The biological basis for the increased AKI risk likely relates to the essential role of zinc in cellular repair mechanisms and stress response pathways (35, 36). Zn deficiency may impair the ability of the kidneys to mount effective responses to oxidative stress, inflammation, and ischemic injury, all of which are central to AKI pathogenesis. This vulnerability may be particularly pronounced in diabetic patients who already face increased baseline oxidative stress and inflammatory burden (37). From a clinical perspective, these findings suggest that zinc status assessment may be particularly valuable for diabetic patients facing procedures or conditions associated with AKI risk, such as contrast exposure, major surgery, or severe illness. The identification of Zn deficiency could prompt enhanced monitoring and potentially prevent interventions in these high-risk scenarios.

The substantial elevation in all-cause mortality risk associated with zinc deficiency likely involves multiple pathways beyond renal function. Zinc deficiency impairs immune function, wound healing, and cellular repair mechanisms, all of which could contribute to increased vulnerability to infectious complications, cardiovascular events, and other life-threatening conditions that are common in patients with diabetes. The central role of the kidney in maintaining overall homeostasis means that zinc deficiency-related renal dysfunction could cascade into systemic complications affecting multiple organ systems. The temporal pattern of mortality risk, with particularly pronounced effects in the short-term follow-up period, suggests that zinc deficiency may serve as a marker of overall physiological vulnerability rather than simply a slow-acting risk factor. This pattern has important implications for clinical care, suggesting that the identification of zinc deficiency should prompt comprehensive assessment and potentially urgent intervention rather than routine long-term management.

The finding that patients with shorter diabetes duration showed more pronounced associations between zinc deficiency and DKD suggests that the protective effects of zinc may be most critical during the earlier phases of diabetic disease progression. There are several possible explanations for this pattern. Early in diabetes progression, when compensatory mechanisms remain intact, an adequate zinc status may be crucial for maintaining protective pathways that prevent initial renal injury. Once advanced diabetic changes occur, the contribution of zinc deficiency may become less apparent against the background of established pathophysiological processes. Alternatively, patients with longer diabetes duration may have adapted to zinc deficiency or may be receiving treatments that modify the effects of zinc. The clinical implications of this finding are significant for preventive care strategies. These results suggest that zinc status assessment may be the most valuable early in diabetes management, potentially serving as a tool for identifying patients who would benefit from intensified early intervention. This could inform the development of risk stratification algorithms that incorporate zinc status alongside traditional factors such as HbA1c and blood pressure. The lack of significant effect modification by other clinical characteristics, including age, sex, and comorbidities, suggests that zinc deficiency represents a broadly applicable risk factor rather than one confined to a specific patient subtype. This consistency strengthens the case for considering zinc status assessment across diverse diabetic populations rather than limiting screening to high-risk groups.

Our study makes several novel contributions to the existing literature. First, to our knowledge, this is the largest real-world analysis to demonstrate that zinc deficiency independently predicts new-onset DKD in patients with T2DM. Second, the stronger associations observed among individuals with shorter diabetes duration suggest that zinc deficiency may confer early vulnerability, highlighting the importance of timely assessment. Third, the absence of an association with glycemic control outcomes supports the notion that zinc deficiency exerts a direct renal effect, rather than merely reflecting poor metabolic control. Together, these findings provide new insights into zinc deficiency as both a biomarker and a potential target for early intervention in diabetic populations.

In current study, overweight/obesity was associated with a lower risk of DKD (HR 0.76), a finding consistent with the so-called “obesity paradox” observed in renal research (38). This paradox may reflect protective metabolic reserves, differential inflammatory profiles, or selective survival bias in patients with higher body mass index. While our findings add to this body of literature, they should be interpreted cautiously, as residual confounding cannot be excluded.

From a clinical perspective, these findings highlight the potential value of routinely assessing zinc status in patients with T2DM, particularly during the early stages of disease. Although causal inference cannot be established from our retrospective design, the consistent associations across renal outcomes and mortality suggest that zinc deficiency may represent a modifiable risk factor. Given the low cost and favorable safety profile of zinc supplementation, targeted correction of deficiency could serve as a pragmatic adjunct to current preventive strategies for DKD. Prospective randomized trials are warranted to determine whether zinc supplementation can directly reduce the risk of DKD and related complications, but our results provide a rationale for heightened clinical awareness and monitoring of zinc deficiency in diabetic populations.

Several limitations of this study should be considered when interpreting our findings. First, the retrospective observational design precludes definitive causal inferences, and unmeasured confounding factors may have influenced the observed associations. While our propensity score matching approach controlled for numerous measured variables, residual confounding from unmeasured factors, such as dietary patterns, socioeconomic status, or genetic factors affecting zinc metabolism, cannot be excluded. Second, the use of ICD-10 coding for outcome ascertainment may introduce misclassification bias, particularly for outcomes such as DKD, where coding practices may vary across institutions. Third, our study population was derived from healthcare systems with electronic health records, which may not be representative of all diabetic populations, particularly those with limited access to healthcare. Additionally, the indication for zinc testing in clinical practice may introduce a selection bias because patients undergoing zinc testing may differ systematically from those who do not receive such testing. Fourth, the study design did not capture information regarding zinc supplementation timing, dosing, or adherence, which limited our ability to assess whether zinc deficiency is a modifiable risk factor. Finally, our follow-up period, while adequate for detecting short-to medium-term outcomes, may not capture the full spectrum of long-term complications associated with zinc deficiency in patients with diabetes. In addition, because the TriNetX platform does not provide access to raw patient-level laboratory data, we were unable to present the detailed distribution of serum zinc levels or assess dose–response correlations with clinical outcomes. Our analyses were therefore limited to dichotomous group comparisons based on predefined zinc thresholds.

## 5 Conclusion

In this cohort study, zinc deficiency was identified as an independent predictor of new-onset DKD and adverse renal outcomes in patients with T2DM. These findings highlight the potential value of routine zinc screening for early risk stratification and management in diabetes care. Future prospective and interventional trials are warranted to confirm causality and evaluate whether zinc supplementation can mitigate the risk of DKD progression.

## Data Availability

The raw data supporting the conclusions of this article will be made available by the authors, without undue reservation.
