# Supplementary material for: Zinc deficiency predicts new-onset diabetic kidney disease in type 2 diabetes: a retrospective cohort study
Source: Front Nutr. 2025 Sep 24;12:1653151. doi: 10.3389/fnut.2025.1653151 (PMC12504847; doi:10.3389/fnut.2025.1653151)
Supplement: Supplementary file 1 [file Table_1.docx]

**Supplemental Table 1**. Subgroup analyses of association between zinc deficiency and risk of diabetic kidney disease (DKD) at 2-Year Follow-Up

| Subgroup analysis | Number of each group | HR (95% CI) | P-value^﹡^ | P for interaction^﹡^ |
| --- | --- | --- | --- | --- |
| Sex |  |  |  |  |
| Male | 2,630 | 1.41 (1.09-1.82) | 0.010 | Reference |
| female | 6,880 | 1.26 (0.99-1.60) | 0.057 | 0.534 |
| Age |  |  |  |  |
| 18-50 years | 2,921 | 1.39 (0.85-2.27) | 0.188 | Reference |
| >50 years | 7,202 | 1.33 (1.11-1.59) | 0.002 | 0.875 |
| Anemia |  |  |  |  |
| Yes | 2,099 | 1.57 (1.08-2.28) | 0.019 | Reference |
| No | 8,093 | 1.29 (1.07-1.56) | 0.008 | 0.403 |
| Hemoglobin A1c ≥7% | |  |  |  |
| Yes | 3,538 | 1.33 (1.04-1.70) | 0.021 | Reference |
| No | 6,670 | 1.34 (1.06-1.69) | 0.013 | 0.965 |
| Hypertension |  |  |  |  |
| Yes | 6,773 | 1.42 (1.17-1.72) | <0.001 | Reference |
| No | 3,417 | 1.75 (1.22-2.51) | 0.002 | 0.361 |
| Duration of T2DM |  |  |  |  |
| <5 years | 7,596 | 1.65 (1.35-2.02) | <0.001 | Reference |
| ≥5 years | 3,181 | 1.07 (0.79-1.45) | 0.667 | 0.016 |
| Dyslipidemia |  |  |  |  |
| Yes | 5,762 | 1.27 (1.03-1.57) | 0.023 | Reference |
| No | 4,419 | 1.35 (1.01-1.80) | 0.040 | 0.742 |
| Use of GLP-1 RAs or SGLT2is | | |  |  |
| Yes | 2,454 | 1.24 (0.88-1.75) | 0.218 | Reference |
| No | 7,736 | 1.32 (1.09-1.60) | 0.005 | 0.754 |

HR: hazard ratio; CI: confidence interval; T2DM: Type 2 diabetes mellitus; GLP-1 Ras: glucagon-like peptide-1 receptor agonists; SGLT2is: sodium-glucose cotransporter-2 inhibitors;﹡P<0.05 was considered significance
